# 

**Published:** 1880-01

**Authors:** 


### Table of Contents.

### ORIGINAL COMMUNICATIONS.

Sciatica Relieved by Galvanism. By H. L. Wilson, M.D., Atlanta, Ga.......... 579
Carbolic Acid—Its Actions and Therapeutic Uses. By J. G. Westmoreland, M.D., Atlanta, Ga.......... 580

### REPORTS OF SOCIETIES.

The New York Academy of Medicine;.......... 584
Philadelphia County Medical Association.......... 593

### SELECTIONS.

Morbid Impulses.......... 596
Compression and Immobilization Principal Factors in Surgery.......... 600
Hemorrhages from the Lungs.......... 604
A Pseudo Tumor of the Abdomen.......... 611
Some Affections of the Upper Air-passages.......... 614
A Case of Traumatic Tetanus—Recovery.......... 620
Absorbtion.......... 623

### EDITORIAL.

In Memoriam.......... 629
Meterological Report for December, 1879.......... 634

### BIBLIOGRAPHICAL.

Walsh's Physicians' Handy Ledger.......... 631
Health Primers.......... 632
First Lines of Therapeutics.......... 632
A Ministry of Health, and other Addresses.......... 632
Memorial Oration in Honor of Ephraim McDowell.......... 632
A Biographical Dictionary of Cotemporary American Physicians and Surgeons.......... 633
Medical Chemistry.......... 633

### EXCERPTA.

The Cause of Malarial Fever.......... 635
A Case of Membranous Croup, with Complete Laryngeal Cast.......... 636
Chrysophanic Acid in Psoriasis and other allied Skin Diseases.......... 638
Urticaria—its Treatment by Belladonna.......... 639
An Outside Description of Hay Asthma.......... 639
The Physiology of Sweating.......... 640
Battey's Operation in Germany.......... 641
Sir Thomas Watson, M.D., on Animal Vaccination.......... 641
Menthol, an Anti-neuralgic.......... 642

